# Role of Oxidative Stress in Drug-Induced Kidney Injury

**DOI:** 10.3390/ijms17111826

**Published:** 2016-11-01

**Authors:** Keiko Hosohata

**Affiliations:** Education and Reseearch Center for Clinical Pharmacy, Osaka University of Pharmaceutical Sciences, Osaka 569-1094, Japan; hosohata@gly.oups.ac.jp; Tel.: +81-72-690-1271

**Keywords:** acute kidney injury, acute interstitial nephritis, acute tubular necrosis, oxidative stress, early biomaker, vanin-1

## Abstract

The kidney plays a primary role in maintaining homeostasis and detoxification of numerous hydrophilic xenobiotics as well as endogenous compounds. Because the kidney is exposed to a larger proportion and higher concentration of drugs and toxins than other organs through the secretion of ionic drugs by tubular organic ion transporters across the luminal membranes of renal tubular epithelial cells, and through the reabsorption of filtered toxins into the lumen of the tubule, these cells are at greater risk for injury. In fact, drug-induced kidney injury is a serious problem in clinical practice and accounts for roughly 20% of cases of acute kidney injury (AKI) among hospitalized patients. Therefore, its early detection is becoming more important. Usually, drug-induced AKI consists of two patterns of renal injury: acute tubular necrosis (ATN) and acute interstitial nephritis (AIN). Whereas AIN develops from medications that incite an allergic reaction, ATN develops from direct toxicity on tubular epithelial cells. Among several cellular mechanisms underlying ATN, oxidative stress plays an important role in progression to ATN by activation of inflammatory response via proinflammatory cytokine release and inflammatory cell accumulation in tissues. This review provides an overview of drugs associated with AKI, the role of oxidative stress in drug-induced AKI, and a biomarker for drug-induced AKI focusing on oxidative stress.

## 1. Introduction

The kidney is an organ that performs a number of essential functions in the body: the clearance of endogenous waste products, the control of volume status, the maintenance of electrolyte and acid-base balance, and endocrine function. Especially, the metabolism and elimination of administered therapeutic and diagnostic agents as well as environmental exposures are major functions. The kidney is exposed to a larger proportion and higher concentration of drugs and toxins than other organs through the secretion of ionic drugs by tubular organic ion transporters across the luminal membranes of the tubule [1], and through the reabsorption of filtered toxins into the lumen of the tubule. Therefore, renal tubular epithelial cells are at greater risk for injury [2,3]. Indeed, drug-induced kidney injury is a serious problem in clinical practice and accounts for 19%–26% of cases with acute kidney injury (AKI) among hospitalized patients [4]. Moreover, AKI causes a severe condition associated with high probabilities of developing progressive chronic kidney disease or end-stage renal disease, thus leading to high mortality rates [5]. Currently, AKI is defined by the Acute Kidney Injury Network (AKIN) as an absolute increase in Scr levels of at least 0.3 mg/dL or a relative Scr increase of more than or equal to 50% within 48 h [6]. However, in some cases, this definition is not applied. For example, tacrolimus-induced AKI in liver transplant recipients is diagnosed by an increase in Scr level of 50% within a continuous 96 h, because the changes in Scr caused by tacrolimus is gradual and difficult to evaluate according to the AKIN criteria [7]. On the other hand, contrast-induced AKI is diagnosed by an early increase in Scr (within 12 h) [8].

## 2. Drugs Responsible for AKI

AKI includes acute tubular necrosis (ATN) and acute interstitial nephritis (AIN). Table 1 shows commonly prescribed drugs which are known to cause AKI, which is due to ATN or AIN.

As for ATN, the renal proximal tubule is commonly damaged by several drugs such as cisplatin [9], aminoglycosides (gentamycin, kanamycin, streptomycin, and tobramycin) [41], amphotericin B [18], antiviral agents (adefovir, cidofovir, and tenofovir) [20], radiocontrast [21], and bisphosphonate [25]. Pathologic characteristics are severe tubular injury including luminal ectasia, marked cytoplasmic simplification, cytoplasmic eosinophilia, loss of brush border, and dropout of tubular epithelia.

AIN is another common cause of AKI. In patients with AKI, approximately 15% are proven to be due to AIN by biopsy [42]. AIN represents an abundant immune response to an exogenously administered medication or toxins. Pathologic characteristic is a diffuse infiltration of lymphocytes, monocytes, plasma cells, and eosinophils into the interstitial compartment. Occasional focus of tubulitis is observed. Many studies show that there is no correlation between the type of offending drug and the histologic findings [43,44]. Generally, renal manifestations of AIN occur with an average delay of approximately ten days [45]. Extrarenal manifestations that indicate a systemic reaction, such as skin eruptions, eosinophilia, and fever, also may occur. Among drugs responsible for AIN, antibiotic agents are most common; however, nonsteroidal anti-inflammatory drugs (NSAIDs) and proton pump inhibitors (PPIs) are also offenders. Recently, PPIs have become one of the most common causes of AIN [46]. In a large nested cohort study, the unadjusted odds ratio for AIN was 5.16 for current versus past PPI use [47]. This effect was obvious in the elderly. This is because of an increased susceptibility in the aging kidney, and because of a higher intake of medications in these patients. Of note, when comparing antibiotic-AIN with PPI-AIN in the elderly, those with antibiotic-AIN exhibited more severe AKI at the time of biopsy [47].

## 3. Mechanism of AKI

In the setting of ATN, the renal proximal tubular epithelium undergoes a complex series of events involving a temporal progression through the loss of polarity and cytoskeletal integrity, necrosis, and apoptosis [48,49,50,51]. Subsequently, necrosis induces inflammation. Necrotic cells release danger-associated molecular patterns (DAMPs) and alarmins from several intracellular compartments. DAMPs are molecules with other proinflammatory functions under normal conditions that turn into danger signals only once being released by cell death and by alerting the innate immune system via a group of pattern recognition receptors (PRRs) on the surface or inside other cells. By contrast, alarmins are a heterogeneous group of preformed proinflammatory molecules that are released by cell death from stores inside the cell [52,53]. The release of DAMPs and alarmins induces inflammation, which implies the recruitment of cytokine-producing leukocytes into the peritubular interstitium. Inflammation accelerates tubular injury [54] and involves potential triggers of necroptosis such as TNF-α [55]. In turn, TNF-α and other cytokines drive necroptosis as a secondary cell death category contributing to tubular necrosis and renal dysfunction. This sets up the auto-amplification loop of necroinflammation [56].

Another mechanism underlying ATN is oxidative stress. Proximal tubular toxicity develops due to direct nephrotoxic effects such as mitochondrial dysfunction, lysosomal hydrolase inhibition, phospholipid damage, and increased intracellular calcium concentration, leading to formation of reactive oxygen species (ROS) with injurious oxidative stress. For example, cisplatin, which induces ATN, invokes oxidative stress, and its pathological conditions under which ROS generates are associated with three mechanisms. First, cisplatin is actuated into a highly reactive form, which can rapidly react with thiol-containing molecules including glutathione (GSH), a well-recognized cellular antioxidant [57,58]. The depletion or inactivation of GSH and related antioxidants leads to the accumulation of endogenous ROS within the cells. It activates signaling pathways, mitogen-activated protein kinase (MAPK), P53 and possibly P21, leading to renal tubular cell death. Subsequently, ROS contribute to the fibrotic process either directly or indirectly via enhanced inflammation. Fibrosis and inflammation itself might feedback to the pathway and further increase ROS formation or stimulate the production of cytokines and growth factors. Second, cisplatin may induce mitochondrial dysfunction and increase ROS production via its disrupted respiratory chain [59]. The role of mitochondrial production of ROS in cisplatin-induced renal injury was further indicated by the cytoprotective effects of mitochondria-localized manganese superoxide dismutase [60]. Interestingly, in the same study, expression of catalase in mitochondria did not have significant protective effects, suggesting that superoxide, and not hydrogen peroxide, may be the major injurious oxidant species generated by mitochondria. Finally, cisplatin may induce ROS formation in the microsomes via the cytochrome P450 (CYP) enzymes. In CYP2E1-null mice, cisplatin-induced ROS accumulation was attenuated, as was renal injury [61]. Similarly, aminoglycosides-induced AKI is involved in oxidative stress. Accumulation of high concentrations within lysosomes and release into the cell cytoplasm promotes phospholipid membrane interruption, oxidative stress, and mitochondrial injury, which cause proximal tubular cell apoptosis and necrosis, leading to AKI.

The mechanism underlying AIN is not completely understood. AIN represents an exuberant host immune response to an exogeneously administered medication or toxin. A proposed mechanism is that absorption of various plasma proteins and molecules by tubular cells causes secretion of chemotactic and inflammatory mediators in the interstitium. It has been reported that nuclear factor-kappa B (NF-κB), a protein complex that regulates DNA transcription and upregulates inflammatory mediators, is overexpressed in the kidneys of proteinuric animals [62,63,64,65]. Increased trafficking of protein has been seen to upregulate RANTES (regulated on activation normal T cell expressed and secreted) production which is a chemoattractant molecule stimulated by NF-κB [66]. The inhibition of NF-κB has been shown to reduce cortical tubulointerstitial injury in rat models [67].

## 4. Oxidative Stress and Vanin-1 as a Potential Biomarker for Drug-Induced ATN

In the development of AKI (especially ATN), ROS and subsequent oxidative stress are largely involved. Generally, ROS are produced as a part of normal cellular function. For example, superoxide anion, the most potent ROS compound, has several cellular sources and is generated as a natural by-product of the electron transport chain in mitochondria. However, under pathological conditions, the uncoupling of oxidative phosphorylation and loss of mitochondrial membrane integrity induce excessive ROS production from the respiratory chain, especially at Complex I and III. Thus, oxidative stress occurs as a result of the increased activity of free radical-producing enzymes, the decreased activity of free radical-removing enzymes, and insufficient levels of antioxidants. In the meantime, mitochondria are also a critical target of the damaging effects of ROS. Oxidative damage leads to mitochondrial dysfunction and a loss of mitochondrial membrane, triggering mitochondrial permeability transition (MPT) and/or the release of proapoptotic proteins like cytochrome c to induce cell death [68].

**Figure 1:**
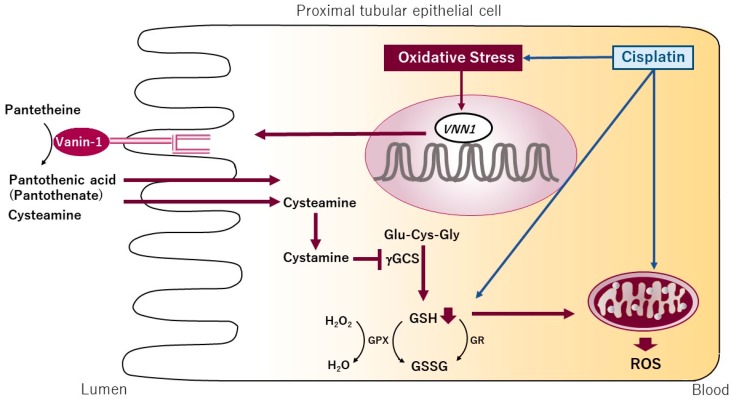
Schematic diagram of the postulated vanin-1 pathway in renal tubular epithelial cells in response to oxidative stress. This overview is based on the works of Dupre et al. [75] and Pitari et al. [72]. An inciting drug (e.g., cisplatin) induces generation of free radical species. Although reactive oxidative stress (ROS) has a positive modulatory role, excessive ROS or inadequate capability of antioxidant scavengers leads to oxidative stress. In the presence of oxidative stress, antioxidant response-like elements within the promoter region of *VNN*1 act as stress-regulated targets and enhance *VNN*1 expression. More cysteamine is produced from hydrolysis of pantetheine; cysteamine is then converted to cystamine, which is an inhibitor of γ-glutamylcytein synthetase (γ-GCS), the rate-limiting enzyme of glutathione (GSH) synthesis. Consequently, GSH stores decrease and subsequently intensifies the oxidative stress, producing more inflammatory cytokines and chemokines. GPX: glutathione peroxidase; GR: glutathione reductase; GSSG: glutathione disulfide.

Considering that a major mechanism of drug-induced AKI (especially ATN) is oxidative stress, it is reasonable to focus on biomarkers that are involved in oxidative stress. Thus, we prepared human primary renal cells [69], and exposed them to organic solvents with nephrotoxicity such as allyl alcohol, chloroform, ethylene glycol, formaldehyde, and phenol, which are known to induce oxidative stress. Next, we extracted total RNA from the cells and analyzed the data at the probe level (CEL files) with GeneSpring GX10 software (Agilent Technologies, Santa Clara, CA, USA) [69], and a novel potential biomarker for AKI (especially ATN), vanin-1 (*VNN1*), which is associated with oxidative stress, was found [70]. Vanin-1 (70 kDa), an epithelial glycosylphosphatidylinositol (GPI)-anchored to cell membrane with pantetheinase activity [71,72], is a tissue sensor for oxidative stress. We validated the increase in its mRNA expression in human proximal tubular cell line, HK-2 cells exposed to organic solvents [70]. In line with our data, Yoshida et al. [73] showed that renal vanin-1 increased about 2.7-fold after renal ischemia-reperfusion in rats, a renal injury model that causes oxidative stress. This means that vanin-1 reflects the activation of pathway of oxidative stress. Furthermore, Berruyer et al. [74] reported that the transcription of *VNN1* is regulated by oxidative stress. A schematic presentation of the postulated vanin-1 pathway is shown in Figure 1.

In the presence of oxidative stress, antioxidant response-like elements within the promoter region of *VNN1* act as stress-regulated targets and enhance *VNN1* expression. More cysteamine is produced from hydrolysis of pantetheine. Thus, cysteamine is converted to cystamine, which is an inhibitor of γ-glutamylcysteine synthetase (γGCS), the rate-limiting enzyme of glutathione synthesis [75]. In *VNN1*−/− mice, which lack cysteamine in tissues, it exhibited resistance to oxidative stress induced by whole-body gamma-irradiation and showed a higher γGCS activity and consequently elevated endogenous stores of GSH, the most potent cellular antioxidant in tissue. This elevated GSH level is correlated with lower ROS concentrations and oxidative damage in tissue and is linked to the survival of animals exposed to stress [74]. These findings for oxidative stress responses supports the reports based on experiments on infection or drug-induced intestinal inflammatory models, where *VNN1*−/− mice display downregulated inflammation [76].

Although *VNN1* transcripts are ubiquitously expressed in mouse organs, the highest levels of *VNN1* mRNAs are found in the kidney where the tubular epithelial cells selectively express the *VNN1* transcripts, but not glomeruli [72]. This expression pattern was confirmed using the anti-vanin-1 antibody, which detected the molecule at the brush border of kidney tubular cells [72]. In line with this report, we found that vanin-1 localized in renal tubules, but not glomeruli localized in the nephrotoxicant-induced renal tubular injury [70].

The physiologic implication of vanin-1 is the recycling of pantothenic acid (vitamin B5, pantothenate). Pantetheinase hydrolyzes one of the amide bonds of pantetheine recycling pantothenic acid (vitamin B5, pantothenate) and releasing cysteamine [75]. Pantothenate is present in food mostly as CoA, which cannot be directly absorbed through enterocytes, whereas pantothenate freely diffuses across the epithelial barrier. Thus, one might speculate that conversion of CoA into pantothenate requires an extracellular, membrane-bound pantetheinase activity capable of recycling pantothenate in the gut. As with the salvage of vitamin B5 in the gut, it is speculated that the presence of a pantetheinase activity at the brush border of tubular epithelial cells might play a role in the salvage of vitamin B5.

The mechanism under which vanin-1 is cleavage is still unknown. Classically, the GPI-anchored proteins are easily released from the cell surface by phosphatidylinositol (PI)-specific phospholipase C (PI-PLC) purified from bacteria [77], which has been used for identification and characterization of the GPI-anchored proteins, although the enzyme is not specific for GPI. GPI-specific PLC was isolated from trypanosomes and characterized in detail [78]. Although other GPI-hydrolyzing PLC activities were described in rat liver [79] and mouse brain [80], the enzymes responsible for these activities have not been characterized in detail. In mammals, the only purified and well-characterized GPI-specific phospholipase is a D-type phospholipase (GPI-PLD). GPI-PLD, a 115-kDa protein, is present in large amounts in mammalian plasma and is capable of cleaving the inositol phosphate linkage of GPI-anchored proteins [81]. Recently, the angiotensin-converting enzyme (ACE) has been reported to be associated with the shedding various GPI-anchored proteins from the cell surface [82]. These molecules could be involved in cleavage of vanin-1.

Until now, various biomarkers for AKI have been identified, such as neutrophil gelatinase-associated lipocalin (NGAL) and kidney injury molecule-1 (KIM-1). KIM-1 is a type-1 cell membrane glycoprotein upregulated in dedifferentiated proximal tubular epithelial cells [83]. Its ectodomain was shed and could be quantitated in the urine following kidney injury in a rodent model of cisplatin-induced AKI [84]. On the other hand, NGAL expression is induced in epithelial cells upon inflammation or malignancy. The expression of NGAL has been shown to be upregulated in the kidney proximal tubular cells and urine in a murine model following ischemic or cisplatin-induced AKI [85]. Importantly, we showed that the urinary concentration of vanin-1 elevated before the conventional markers such as serum creatinine, urinary *N*-acetyl-β-d-glucosaminidase (NAG), or both increased in rats with a nephrotoxicant [70], and cisplatin [86] induced renal tubular injury in the time-course analyses. Furthermore, urinary vanin-1 was shown to be more predictive of the decline in eGFR after the dosing of cisplatin compared with KIM-1, NGAL, and NAG in patients with urothelial carcinoma [87].

The limitation of urinary vanin-1 as a potential biomarker is as follows: many hospitalized patients are likely to be receiving these drugs due to their systemic inflammation of various etiologies; therefore, it is difficult to differentiate between systemic oxidative stress (e.g., due to sepsis) and oxidative stress in the kidney (e.g., due to cisplatin).

## 5. Conclusions

Urinary vanin-1 could be a useful biomarker for the detection of drug-induced ATN focusing on oxidative stress. On the other hand, vanin-1 remains to be tested for drugs causing AIN. In addition, there are other mechanisms of drug-induced AKI. Further studies are needed to exploit more favorable biomarkers for drug-induced AKI.

## Figures and Tables

**Table 1:** Drugs responsible for acute kidney injury.

Type of Damage	Drug	Pharmacological Class	References
ATN	Cisplatin	Chemotherapeutic agents	[9]
	Ifosfamide	Chemotherapeutic agents	[10]
	Pemetrexed	Chemotherapeutic agents	[11,12]
	Gentamycin	Antibiotics	[13,14]
	Kanamycin	Antibiotics	[15]
	Streptomycin	Antibiotics	[15]
	Tobramycin	Antibiotics	[15]
	Colistin	Antibiotics	[16,17]
	Amphotericin B	Antifungal	[18]
	Foscarnet	Antiviral agents	[19]
	Adefovir	Antiviral agents	[20]
	Cidofovir	Antiviral agents	[20]
	Tenofovir	Antiviral agents	[20]
	Iopromide	Radiocontrast	[21]
	Cyclosporine A	Immunosuppressive	[22]
	Tacrolimus	Immunosuppressive	[23]
	Pamidronate	Bisphosphonate	[24]
	Zoledronic acid	Bisphosphonate	[25]
	Acetaminophen	Analgesic	[26]
AIN	Penicillins	Antibiotics	[27,28]
	Cephalosporins	Antibiotics	[29,30]
	Quinolones	Antibiotics	[31,32,33]
	Vancomycin	Antibiotics	[34,35]
	Rifampicin	Antibiotics	[36,37]
	NSAIDs	Anti-inflammatory, analgesic, antipyretic	[38]
	Omeprazole	Proton pump inhibitors	[39]
	Ipilimumab	Immune check point inhibitors	[40]
	Nivolumab	Immune check point inhibitors	[40]

ATN: acute tubular necrosis; AIN: acute interstitial nephritis; NSAIDs: nonsteroidal anti-inflammatory drugs.

## Abbreviations

AKI	acute kidney injury
ATN	acute tubular necrosis
AIN	acute interstitial nephritis
ROS	reactive oxygen species
GSH	glutathione
